# Alleviating the burden of depression: a simulation study on the impact of mental health services

**DOI:** 10.1017/S204579602400012X

**Published:** 2024-04-02

**Authors:** M. Wilhelm, S. Bauer, J. Feldhege, M. Wolf, M. Moessner

**Affiliations:** 1 Center for Psychotherapy Research, Heidelberg University Hospital, Heidelberg, Germany; 2 Institute of Psychology, Heidelberg University, Heidelberg, Germany; 3 German Center for Mental Health (DZPG), Partner site Mannheim/Heidelberg/Ulm, Germany; 4 Asklepios Science & Research, Research Institute, Hamburg, Germany; 5 Department of Psychology, University of Zurich, Zürich, Switzerland

**Keywords:** depression, health service research, mental health, psychotherapy, social and political issues

## Abstract

**Aims:**

Depressive disorders are ranked as the single leading cause of disability worldwide. Despite immense efforts, there is no evidence of a global reduction in the disease burden in recent decades. The aim of the study was to determine the public health impact of the current service system (status quo), to quantify its effects on the depression-related disease burden and to identify the most promising strategies for improving healthcare for depression on the population level.

**Methods:**

A Markov model was developed to quantify the impact of current services for depression (including prevention, treatment and aftercare interventions) on the total disease burden and to investigate the potential of alternative scenarios (e.g., improved reach or improved treatment effectiveness). Parameter settings were derived from epidemiological information and treatment data from the literature. Based on the model parameters, 10,000,000 individual lives were simulated for each of the models, based on monthly transition rates between dichotomous health states (healthy vs. diseased). Outcome (depression-related disease burden) was operationalized as the proportion of months spent in depression.

**Results:**

The current healthcare system alleviates about 9.5% (95% confidence interval [CI]: 9.2%–9.7%) of the total disease burden related to depression. Chronic cases cause the majority (83.2%) of depression-related burden. From a public health perspective, improving the reach of services holds the largest potential: Maximum dissemination of prevention (26.9%; CI: 26.7%–27.1%) and treatment (26.5%; CI: 26.3%–26.7%) would result in significant improvements on the population level.

**Conclusions:**

The results confirm an urgent need for action in healthcare for depression. Extending the reach of services is not only more promising but also probably more achievable than increasing their effectiveness. Currently, the system fails to address the prevention and treatment of chronic cases. The large proportion of the disease burden associated with chronic courses highlights the need for improved treatment policies and clinical strategies for this group (e.g., disease management and adaptive or personalized interventions). The model complements the existing literature by providing a new perspective on the depression-related disease burden and the complex interactions between healthcare services and the lifetime course.

## Introduction

Depression is associated with substantial consequences for the individuals affected, their social network, the public healthcare system and society as a whole (Murray *et al.*, 2012; GBD 2016 Disease and Injury Incidence and Prevalence Collaborators, 2017). The Global Burden of Diseases, Injuries, and Risk Factors Study (GBD) 2019 found depressive disorders causing the largest proportion of the burden of mental disorders, contributing up to 37.3% of overall mental disability burden (GBD 2019 Mental Disorders Collaborators, 2022). Also, with an estimated 4.4% of the world’s population affected, depressive disorders are the single leading cause of disability (World Health Organization, 2017).

The challenges in reducing the disease burden are manifold as depression is not only a common but also a complex mental health condition with heterogeneous and often episodic or long-term courses. Approximately, half of untreated episodes remit spontaneously within a year (Whiteford *et al.*, 2013), but in 20%–30% of cases, an episode can last for 2 years or longer (Jobst *et al.*, 2016). After suffering from a first episode, about 40%–60% of affected individuals experience at least one additional episode (Bockting *et al.*, 2015), and the risk of subsequent episodes increases with each episode (Solomon *et al.*, 2000). Overall, both the number and duration of episodes are predictors of the further course of depression (Moriarty *et al.*, 2022). Lifelong consequences include severe impairment of quality of life and excess mortality (Schneider *et al.*, 2019).

Numerous evidence-based psychological interventions for the prevention (van Zoonen *et al.*, 2014), treatment (Cuijpers *et al.*, 2019) and aftercare (Biesheuvel-Leliefeld *et al.*, 2015) of depression have been introduced. However, despite significant progress over the past decades, the effectiveness of even the best available interventions is limited (Ormel and Emmelkamp, 2023). For example, non-response and partial response are relatively common in psychotherapy with only about 60% of patients not meeting criteria for major depression at the end of treatment (Cuijpers *et al.*, 2014). Similarly, preventive interventions and maintenance treatment have limited effects with an estimated reduction of illness onset and recurrence by 21% (van Zoonen *et al.*, 2014) and 36% (Biesheuvel-Leliefeld *et al.*, 2015), respectively.

A second and equally important challenge in the care for depression refers to the fact that only a minority of affected individuals receive adequate help (Thornicroft *et al.*, 2017; Wang *et al.*, 2007). An intervention’s public health impact is defined as the product of its effectiveness and reach (effectiveness*reach; Glasgow *et al.*, 2003), i.e., highly effective interventions have only little impact on the population level when they only reach few people and less effective interventions may actually have a higher impact when they reach a larger part of the population. Factors contributing to the significant treatment gap in depression include both organizational barriers (e.g., limited availability and long waiting times) as well as individual barriers (e.g., lack of mental health literacy and stigma) (Thornicroft *et al.*, 2017).

Although an urgent need for action related to healthcare for depression has been pointed out for many years (Murray and Lopez, 1996; Üstün *et al.*, 2004), there is no evidence of a global reduction in the depression-related disease burden since 1990 (GBD 2019 Mental Disorders Collaborators, 2022). To move forward, it is of utmost importance to better understand the complex interplay of different healthcare services, to identify structural bottlenecks and to determine the potential of specific strategies aiming to reduce the illness burden on a population level. To that end, statistical modelling can be used to inform and guide decision-making processes (Kazdin and Blase, 2011; Lokkerbol *et al.*, 2014; Moessner and Bauer, 2017; Vos *et al.*, 2004).

Therefore, the aims of this simulation study were first to quantify the overall extent to which current healthcare including prevention, treatment and aftercare reduces the depression-related disease burden and second to estimate the impact that changes in specific areas of healthcare would have on this reduction.

## Method

A Markov model was developed to quantify the impact of various healthcare scenarios (i.e., current healthcare versus modified healthcare scenarios) on the disease burden of depression over the lifetime on a population level. Parameter settings were derived from the literature and selected in an iterative process.

### Model parameters

The model included the following parameters: Incidence rates for depression, taking into account age and sex (the overall yearly incidence rate of depressive disorders was 1.14%; Gerste and Roick, 2016), spontaneous remissions (23% within 3 months, 32% within 6 months and 53% within 1 year; Whiteford *et al.*, 2013) and sex-specific and depression-related excess mortality rates (Schneider *et al.*, 2019; Statistisches Bundesamt, 2020a, 2020b). In addition, the model considered individuals’ lifetime history of depression. Since the number of episodes is related to the probability of recurrence, the probability of recurrence within the model increases with the number of previous episodes (16% increase per episode; Solomon *et al.*, 2000). For example, the monthly transition probability from healthy to diseased after the first month in remission increases from 2.36% (one prior episode) to 2.74% (two prior episodes). Furthermore, the risk of recurrence progressively decreases with an individual’s time spent in remission. Parameters with known international variability (incidence rates, mortality rates, reach of treatment, waiting time for treatment, maximum duration of treatment and aftercare) were estimated based on data from Germany (Bundespsychotherapeutenkammer, 2018; Busch *et al.*, 2013; Gerste and Roick, 2016; Rommel *et al.*, 2017; Statistisches Bundesamt, 2020b, 2020a). We assumed an average waiting time of 5 months (*SD* = 1 month) from seeking to actually receiving treatment and a maximum duration of psychotherapy and aftercare of 24 months (Bundespsychotherapeutenkammer, 2018) and 12 months (Bundesärztekammer *et al.*, 2022), respectively.

Parameters related to the effectiveness and reach of services (i.e., prevention, non-pharmacological treatment/psychotherapy and aftercare) are displayed in Table 1. For each of these parameters, 5% increments up to 100% were simulated to estimate the impact of various healthcare scenarios (absolute percentages).

**Table 1.** Selection of model parameters

Parameter	Definition	Setting
Effect-prevention^a^	Preventive interventions reduce the first onset of depression by 21%.	.21
Reach-prevention	Proportion of target population receiving prevention measures estimated to be 5%.	.05
Effect-treatment^b^	Proportion of patients who do not meet criteria for depression after treatment is 62%.	.62
Reach-treatment^c^	Proportion of depressed who seek treatment within a year is about 33%.	.33
Effect-aftercare^d^	Aftercare interventions reduce the risk of recurrence by 36%.	.36
Reach-aftercare	Proportion of those treated who receive aftercare estimated to be 5%.	.05

a van Zoonen *et al.* (2014).

b Cuijpers *et al.* (2014).

c Rommel *et al.* (2017).

d Biesheuvel-Leliefeld *et al.* (2015).

### Transition probabilities

Based on the model parameters, monthly transition probabilities between healthy and diseased states were calculated (see supplementary material for full code).

Example: Assuming about 53% of individuals remit spontaneously over the course of a year (Whiteford *et al.*, 2013), the converted monthly transition probability is 6.098% (
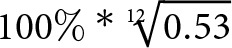
). Acute treatment impacts remission positively and may contribute to shorter episode duration, resulting in a 71.3% chance 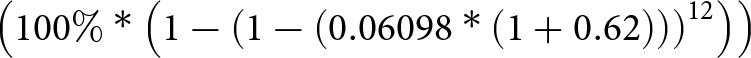 for the individual to remit during receiving 12 months of treatment. However, the individual must first decide to seek help and then do the wait (delay between help seeking and treatment).

### Primary outcome

Monthly transition rates between healthy and diseased were modelled, and the overall illness burden was defined as the proportion of months in depression. Acknowledging largely varying definitions of these terms in the literature, we used the following pragmatic definitions for the purpose of this study: *Remission* describes the transition from a diseased to a healthy state and *recurrence* describes the transition from a healthy to a diseased state. Furthermore, we defined *chronic courses* as experiencing at least one depressive episode that lasted longer than 24 months or as experiencing three or more depressive episodes. The disease burden was operationalized by the proportion of months spent in depression:

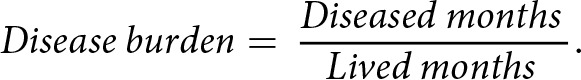


### Procedures and analysis

A simulation of the current healthcare system was conducted to assess the plausibility and validity of the model by predicting life expectancy, the life-time incidence of depression and the average number and duration of depressive episodes. In addition, the extent to which current healthcare reduces the depression-related disease burden was quantified. Subsequently, we estimated the impact that the above-mentioned improvements in reach and effectiveness in specific healthcare settings would have on this reduction.

Monte Carlo simulations were run 1,000 times with 10,000 simulated lives per run, resulting in a total of 10,000,000 simulated lives per model. Based on the 1,000 runs, 95% confidence intervals (CIs) were calculated. All simulations were performed in R (Version 4.1.2; R Development Core Team, 2021) using the packages *doRNG* (Gaujoux, 2020), *doParallel* (Folashade *et al.*, 2022), *dplyr* (Wickham *et al.*, 2022a) and *readr* (Wickham *et al.*, 2022b). Computations were run on the high performance bwUniCluster 2.0 (https://wiki.bwhpc.de/e/BwUniCluster2.0).

## Results

### Model plausibility

The model’s predictions (see Table 2) proved plausible, stable and in line with the literature. Lifetime incidence of depression amounted to 11.4%, and about 45% of depressed individuals received treatment at least once. On average, individuals affected experienced *M* = 2.6 episodes of depression (*SD* = 2.5). Fifty-four percent of those affected experienced recurrence at least once. The average duration of an episode was predicted to be *M* = 14.5 months (*SD* = 10.5) with averages of *M* = 9.4 months (*SD* = 5.8) and *M* = 19.9 months (*SD* = 11.6) for non-chronic and chronic cases, respectively.

**Table 2.** Plausibility check for the current healthcare model on a population level

	Model outcome			Reference value		
*N* = 10,000,000	Total	Female	Male	Total	Female	Male
Life expectancy *M* (*SD*)	80.9 (13.7)	83.3 (12.9)	78.5 (14.1)	–	83.3^a^	78.5^b^
Lifetime prevalence	11.4	13.7	9.0	11.6^c^	15.4^c^	7.8^c^

a Statistisches Bundesamt (2020b).

b Statistisches Bundesamt (2020a).

c Busch *et al.* (2013).

Concerning chronicity, the model indicated that 48.3% of the individuals that were affected by depression (i.e., 550,644 of 1,140,621 depressed cases) experienced a chronic course of illness. More specifically, 34.1% of all individuals with depression suffered from at least one episode lasting longer than 24 months, and 31.9% suffered from at least three depressive episodes. When comparing the totals of diseased months of the non-chronic versus the chronic group, chronic cases accounted for 83.2% of all diseased months.

### Reduction of disease burden

**Figure 1.**
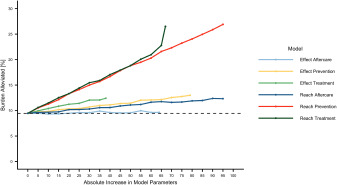
Simulation results for the alternative models (changes in parameter settings) for absolute 5% increments up to an optimal scenario with 100%. The parameter settings for the current system can be found in Table 1.

Figure 1 shows the results of the simulations. The *x*-axis represents parameter increases in absolute 5% increments up to an optimal scenario with 100%, assuming that the other parameters are kept constant. The *y*-axis shows the reduction of the disease burden compared to a scenario without healthcare.

**Figure 2.**
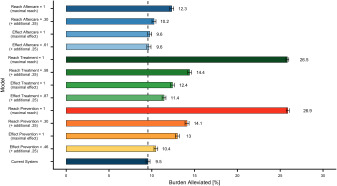
Selection of simulation results for an increase of 25% and to an optimal scenario with 100%. Error bars represent upper and lower CI limits. The dashed line refers to the disease burden alleviated by the current system.

Concerning the first study aim, the results indicate that the current healthcare system alleviates about 9.5% (CI: 9.2%–9.7%) of the disease burden caused by depression (shown in Fig. 2). Concerning the second study aim, the findings show that improving the reach of services holds the greatest potential: Increasing the reach of prevention by 25% (from 5% to 30%) results in 14.1% (CI: 13.9%–14.4%) reduction in the disease burden, i.e., an additional 4.6%. In the hypothetical scenario that the total population participated in prevention (100% instead of 5%), the impact of the healthcare system in reducing the disease burden would almost triple from 9.5% to 26.9% (CI: 26.7%–27.1%).

Similarly, increases of 25% in the reach of treatment (increase in the proportion of depressed individuals seeking treatment within 1 year from 33% to 58%) would result in an additional 4.9% reduction in the population’s disease burden (reduction of 14.4%; CI: 14.2%–14.6%). If every affected individual was to seek treatment (increasing reach of treatment from 33% to 100%; leading to an immediate search for help), up to 26.5% (CI: 26.3%–26.7%) of the total disease burden of depression could be alleviated. Other improvements in the healthcare system could, for example, reduce the disease burden only by an additional 0.1% (effect of aftercare set to 100%: 9.6%; CI: 9.4%–9.9%), an additional 2.9% (effect of treatment set to 100%: 12.4%; CI: 12.2%–12.7%) or an additional 3.5% (effect of prevention set to 100%: 13.0; CI: 12.8%–13.2%).

## Discussion

To the best of our knowledge, this model-based simulation study is the first that quantifies the impact that changes in specific areas of healthcare would have on the disease burden of depression at the population level based on monthly transitions within a lifetime perspective. By using data from Germany as a use case, the model gives conceptual insights into the complex interplay between the nature of depression and the healthcare system, and thus, it provides a valuable basis for discussion and decision-making. Our results also pinpoint the boundaries within which the current system operates; the present situation is far from satisfactory, as the current healthcare system has only little impact on the overall disease burden of depression with an estimated reduction of less than 10%. Based on the findings of this study, the most promising way forward clearly is to focus on widely disseminating (existing) preventive and therapeutic services (i.e., to maximize reach) rather than on enhancing the outcome of prevention and treatment (i.e., to maximize effectiveness). However, large-scale dissemination of interventions is of course only part of the healthcare challenge (Herrman *et al.*, 2022).

Although chronic cases account for over four fifths of the disease burden, these cases are not adequately addressed by the current healthcare system. Given the lifelong course of the disease in many cases, a long-term perspective and more flexible strategies to manage depression are needed (Bockting *et al.*, 2015; Legemaat *et al.*, 2023). During symptom-free periods, low-intensity digital interventions might be applied to prolong periods of well-being and monitor symptoms in order to detect relapses and initiate adequate treatment without delays (Kordy *et al.*, 2016). On a population level, however, the simulated improvements in the aftercare sector will probably still be relatively small, which is to be explained by the interplay of different healthcare sectors. Within the model, the effects of aftercare effect and reach are highly interdependent on the other parameters, i.e., as long as only a minority of affected individuals receive treatment, even in a scenario where aftercare would be available to 100% of affected individuals, only about half (45%) of affected individuals will receive treatment at least once in their life, and not all treatments are successful. These interaction effects reduce possible effects of changes in parameters, especially for interventions in the post-treatment period. Therefore, at the macro level, it is essential for any form of aftercare or long-term disease management to first enhance uptake of initial treatment, i.e., engage as many individuals as possible in the healthcare system. Although some of these considerations might be specific for the German healthcare system, the majority of countries face similar challenges that limit the population-level effectiveness of their current services.

In routine care, i.e., outside of this simulation environment, however, it might be extremely complicated to broadly scale up existing interventions and reach a substantial proportion of unserved individuals. Various barriers stand in the way of providing adequate treatment for the majority of those affected (Andrade *et al.*, 2014; Kazdin, 2017; Thornicroft *et al.*, 2017). These include attitudinal barriers (e.g., stigma or lack of mental health literacy), systemic barriers (e.g., cost of services or political and legal constrains) and other factors such as the limited availability of services, due to the dominant model of treatment delivery still being in-person individual psychotherapy. Again, technological advances (e.g., symptom monitoring, digital interventions, machine learning and AI algorithms) might allow us to deliver part of our mental health services and could potentially free up professional capacities to provide more intense interventions to patients who need them most. For instance, especially in periods of well-being, digital interventions can provide low-intensity, low-cost aftercare or long-term disease management (Bauer *et al.*, 2012; Kordy *et al.*, 2016). In addition to the prevention of future episodes, these kinds of interventions can also detect new episodes and facilitate early treatment uptake in order to shorten the duration of episodes. In addition, an optimized resource allocation using data driven, individualized predictions to support treatment selection decisions (e.g., stratified care; Delgadillo *et al.*, 2022), could increase the efficiency of mental health systems even without improving treatment effectiveness (Cohen and Derubeis, 2018).

### Strengths and limitations

The strength of the presented model is that it quantifies the impact of different healthcare scenarios on the disease burden of depression at the population level. By simulating a lifetime horizon and integrating monthly state transitions, the model does justice to the fluctuating and often lifelong nature of depressive disorders.

At the same time, the model has some limitations that need to be considered. First, the development of the Markov model required several assumptions and simplifications. For example, the available database is quite scarce, e.g., as studies often lack information about type, duration and long-term outcomes of interventions (Kazdin, 2017). Therefore, available epidemiologic and clinical data were extrapolated as well as derived from different studies and healthcare systems. As mentioned above, some specific parameters were based on the German healthcare system and thus might not generalize to healthcare systems in other countries. Further, balancing the trade-off between the appropriateness and complexity of the model, severity of episodes, comorbidities and pharmacotherapy were not included in the model. Second, the model uses a dichotomous outcome (healthy vs. diseased), which only superficially describes the complexity of depression and the suffering of those affected. Third, a cost analysis would add additional value to the study. It would allow to estimate resource requirements and calculate optimal resource allocation from a public health point of view. Yet, this is beyond the scope of this study (for health economic models, see Baumann *et al.*, 2020; Lokkerbol *et al.*, 2014). Despite these limitations, however, the model provides plausible conceptual insights into the complex interplay between the nature of depression and healthcare services.

### Implications

Overall, improving the dissemination and thus the reach of existing interventions are not only more promising to reduce the immense disease burden of depression but probably also more realistic than achieving a substantial increase in the effectiveness of available services. Moreover, reducing the treatment gap and facilitating access to the healthcare system are an important and basic prerequisite for a long-term perspective and adaptive disease-management strategies that might address the needs of patients with chronic conditions. Overall, our findings add empirical data to the discussion and are in line with recent recommendations which underline the need to improve the impact that healthcare systems have on the burden of depression (Herrman *et al.*, 2022).

## Conclusion

The results confirm the urgent need for action in the healthcare for depression as current services reduce the disease burden by less than 10%. Improving the reach of services holds the largest potential. The large proportion of illness burden associated with chronic courses (83%) highlights the need for strategies that are specifically tailored to the needs and challenges in this group, including disease management, and adaptive or personalized long-time intervention strategies. Simulation studies are a valuable tool to inform and guide future decision-making processes.

## Supporting information

Wilhelm et al. supplementary material Wilhelm et al. supplementary material

## Data Availability

The data generated in this study are available on request. All data and scripts needed to perform the simulation model are included in this article and its supplementary material files. Further enquiries can be directed to the corresponding author.

## Supplementary material

The supplementary material for this article can be found at https://doi.org/10.1017/S204579602400012X.
