# Supplementary material for: Alleviating the burden of depression: a simulation study on the impact of mental health services
Source: Epidemiol Psychiatr Sci. 2024 Apr 2;33:e19. doi: 10.1017/S204579602400012X (PMC11022261; doi:10.1017/S204579602400012X)
Supplement: Wilhelm et al. supplementary material [file S204579602400012Xsup001.pdf]

Supplementary Material

Alleviating the Burden of Depression:
A Simulation Study on the Impact of Mental Health Services

Table of contents

R-Script

R-Script for Depression Model....................2

Tables

Table S1: Parameter settings for model variation (set01001.csv)....................8

Table S2: Incidence-, mortality-, and excess mortality-rates (age_tables.csv)....................9

Table S3: Relapse probability for monthly transition from healthy to depressed (relapse.csv)....................11

R-Script for Depression Model

```
# Alleviating the Burden of Depression:
# A Simulation Study on the Impact of Mental Health Services

# Wilhelm, M., Bauer, S., Feldhege, J., Wolf, M., & Moessner, M.
# Center for Psychotherapy Research, Heidelberg
# Contact: Maximilian.Wilhelm@med.uni-heidelberg.de


#  0 - General Comments ---------------------------------------------------
# The Set-name "set01001" sets the seed for the simulation. If further cases are to be calculated in
# parallel, this R-file can be named "set01002", for example.

# Running the code in Section 3-5:
# 1) The settings must be adapted to the operating system.
# 2) The result of the simulation is written to csv files.
#    If complete_data = T (TRUE), then a detailed CSV file is created, in which the individual months of
#    each simulated person are recorded.
#    If complete_data = F (FALSE; default), then a CSV file is created, in which only the summary for the
#    person is stored.

# The concept of long-term depression management (ltdm) is not addressed in the publication, but
# is included in the code for future simulation.
# In the cases analyzed for the publication, the parameters of the management are kept constant and have
# negligible effects on the results.

# 1 - Preparation ---------------------------------------------------------

# Set working directory

# Install and load packages
# Define the list of packages
packages <- c("doRNG", "doParallel", "dplyr", "readr", "snow")

# Check if packages are installed
missing_packages <- packages[!(packages %in% installed.packages()[,"Package"])]

# Install missing packages
if (length(missing_packages) > 0) {
  install.packages(missing_packages, dependencies = TRUE)
}

library(doRNG)      # Version 1.8.6
library(doParallel) # Version 1.0.17
library(dplyr)      # Version 1.1.2
library(readr)      # Version 2.1.4
library(snow)       # Version 0.4-4

# Set a seed for reproducible results
# Composition: name of parameter set: set01001 (set, simulation number 01, set number 001)

Set<-"set01001.csv"

SetName0<-strsplit(Set, "\\.")[[1]][[1]] # Cut the suffix
SetName1<- substring(SetName0,1, nchar(SetName0)-5) # Cut last five digits of file name
SetName<-(paste(match(unlist(strsplit(SetName1, split = "")), letters[1:26]), collapse = ""))
SetNr<-(substring(SetName0,nchar(SetName0)-3)) # Get last five digits of file name
seed<-as.numeric(paste(SetName, SetNr, sep="")) # Assign seed

# Read parameter list for parallel computing from excel
params <- read.csv(Set, sep =";", dec = ",", stringsAsFactors = FALSE)
names(params) <- c("file", "effect_prevention", "reach_prevention",
                   "effect_treatment", "reach_treatment","tlimit_treatment",
                   "effect_aftercare", "reach_aftercare", "tlimit_aftercare",
                   "effect_ltdm", "reach_ltdm","tlimit_ltdm","ltdm_coordination")
params_list <- split(params, sort(as.numeric(rownames(params))))

# Create result file
S_Result_DF <- data.frame("ID" = 0, "gender" = "f", "age_y" = 0,
                          "age_m" = 0, "episode" = 0, "help_seeking" = 0,
```

```
                        "treatment" = 0, "total_depression" = 0, "chronic" = 0,
                        "aftercare" = 0, "ltdm" = 0, "onset" = 0, "s_death" = 0,
                        "persistent" = 0)

S_Result<-S_Result_DF
S_Line<-S_Result_DF

# Load data
age_tables <- read.table("age_tables.csv", header = T, sep = ";", dec = ",")
# Incidence = probability to develop first episode of depression (age & gender; Gerste & Roik, 2015)
# Excess mortality factor due to depression (age & gender; Schneider, 2019)

relapse  <-read.table("relapse.csv", header = T, sep = ";", dec = ",") # Relapse rates  (Solomon, 2000)
names(relapse) <- c("time","E_1","E_2","E_3","E_4","E_5","E_6")

# 2 - Loop function -----------------------------------------------------

loop_f <- function(pars,
                  Num,
                  complete_data = FALSE){

  TS<- c(1,2) # Transition states
  DS<- c(0,1) # Death states

  recovery        <- 0.06098  # Average probability for monthly recovery
  delay_treatment <- 5        # Average delay between help seeking and treatment (waiting time)
  ltdm_en         <- 3        # Definition of recurrent depression
  ltdm_ed         <- 24       # Definition of persistent depression

# Preventive interventions reduce the first onset of depression by effect_prevention
  effect_prevention <- as.numeric(pars$effect_prevention)
# Proportion of population receiving prevention measures is set to reach_prevention
  reach_prevention  <- as.numeric(pars$reach_prevention)
# effect_treatment positively impacts recovery probability
  effect_treatment  <- as.numeric(pars$effect_treatment)
# Proportion of depressed who seek treatment within a year is set to reach_treatment
  reach_treatment   <- 1-(1-as.numeric(pars$reach_treatment))^(1/12)
# Treatment ends after tlimit_treatment
  tlimit_treatment  <- as.numeric(pars$tlimit_treatment)
# Aftercare interventions reduce the risk of recurrence by effect_aftercare
  effect_aftercare  <- as.numeric(pars$effect_aftercare)
# Proportion of those treated who receive aftercare estimated to be reach_aftercare
  reach_aftercare   <- as.numeric(pars$reach_aftercare)
# Aftercare ends after tlimit_aftercare
  tlimit_aftercare  <- as.numeric(pars$tlimit_aftercare)
# Long-term interventions (long-term depression management; ltdm) can contribute to prolongation of
# symptom-free phases (Kordy et al., 2016)
  effect_ltdm       <- as.numeric(pars$effect_ltdm)
# Proportion of those treated who receive ltdm estimated to be reach_ltdm
  reach_ltdm        <- as.numeric(pars$reach_ltdm)
# ltdm ends after tlimit_ltdm
  tlimit_ltdm       <- as.numeric(pars$tlimit_ltdm)
# ltdm could promote reach_treatment & shorten delay_treatment
  ltdm_coordination <- as.numeric(pars$ltdm_coordination)

  Num <- Num # N for each simulation

  createperson = function(nrow=1) {
    ID <- IDX#(c(1:nrow))
    gender <- sample(c("f","m"),1,replace=TRUE,
                   prob = c(42.053/83.019,40.967/83.019))
    state           <- 1 # State birth = healthy
    age_y           <- 0 # Age in years
    age_m           <- 0 # Lived months (loops)
    transition      <- 0 # Change of state
    episode         <- 0 # Number of episodes
    time_depression <- 0 # Count time in depression per episode
    time_stability  <- 0 # Count time in healthy condition after episode (stability of state)
    help_seeking    <- 0 # Help seeking (0 = No, 1 = Yes)
    waiting         <- 0 # Count waiting for treatment
    treatment       <- 0 # Receiving treatment
    treatment_t     <- 0 # Count time receiving treatment
```

```
  total_depression<- 0 # Count total time in depression
  chronic        <- 0 # Indicate chronic Depression (episode duration > 24 months / episode > 2)
  chronic_24      <- 0 # Indicate chronic Depression (episode duration > 24 months)
  aftercare      <- 0 # Receiving aftercare following treatment
  aftercare_t     <- 0 # Backward count person receives aftercare
  ltdm           <- 0 # Long term depression management
  ltdm_t         <- 0 # Backward count person receives ltdm
  df_person      <- dplyr::tibble(ID, gender, state, age_y, age_m,
                              transition, episode, time_depression, time_stability,help_seeking,
                              waiting, treatment, treatment_t, total_depression, chronic,chronic_24,
                              aftercare, aftercare_t, ltdm, ltdm_t)
}

lookup_prob <- function(df, type, age, gender){
  df[df$age == age, paste(type, gender, sep = "_")]
}

### Loop 1: State = 1  - Healthy loop
F1<-function(){
  aux_line <- s_out[nrow(s_out),] # Load the auxiliary line
  age      = aux_line$age_y # Load age
  episode  = aux_line$episode # Load number of episode

  ## Transition probabilities ##
  # Transition to depressed
  p_depressed = if (episode == 0){
    lookup_prob(age_tables, type ="incidence", age, gender)*(1-effect_prevention*reach_prevention)
  }else {relapse[aux_line$time_stability+1,
               dplyr::if_else(aux_line$episode < 5, aux_line$episode+ 1, 7), drop = T]}
  # Effect aftercare
  if (aux_line$aftercare == 1){p_depressed = p_depressed*(1-effect_aftercare)} else {NULL}
  # Effect ltdm
  if (aux_line$ltdm == 1) {p_depressed = p_depressed*(1-effect_ltdm)} else {NULL}

  h_state = sample(TS, 1, replace = T, prob = c (1-p_depressed,p_depressed))

  # Transition to death
  p_death   = lookup_prob(df = age_tables, type = "mortality", age, gender)
  d_state = sample(DS, 1, replace = T, prob = c (1-p_death,p_death))

  # Transition
  transition_state = if(d_state==1){3}else{h_state}



  ## Save new state and prepare next loop ##
  # Indicate state transition
  if(transition_state != aux_line$state) {aux_line$transition = 1} else {NULL}
  aux_line$state = transition_state   # Transition
  aux_line$age_m = aux_line$age_m + 1 # Person ages for one month
  aux_line$age_y = trunc(aux_line$age_m/12, 0) # Age in years
  if(aux_line$episode > 0 && transition_state == 1){aux_line$time_stability = aux_line$time_stability+1} else {NULL}
# Start count time_stability

  if(transition_state != 2){NULL}else{ # If new episode starts, do the following
    aux_line$episode          = episode+1; # Episode + 1
    aux_line$time_depression   = aux_line$time_depression+1; # Start count time in D
    aux_line$total_depression  = aux_line$total_depression+1 # Count total time in D
    aux_line$time_stability    = 0 # Set time in stability to 0
  }
  # Count time in aftercare backwards
  if(aux_line$aftercare_t>=1){aux_line$aftercare_t = aux_line$aftercare_t-1} else {NULL}
  # If time limit aftercare is reached, end aftercare
  if(aux_line$aftercare_t==0){aux_line$aftercare = 0} else {NULL}
  # Count time in ltdm backwards
  if(aux_line$ltdm == 1){aux_line$ltdm_t = aux_line$ltdm_t-1}else{NULL}
  # If time limit ltdm is reached, end ltdm
  if(aux_line$ltdm_t==0){aux_line$ltdm=0; aux_line$waiting=0}else{NULL}
  # Attach the auxiliary line to the dataset (s_out)
  s_out <- dplyr::bind_rows(s_out,aux_line)  }
```

```r
### Loop 2: State = 2  - Depressed loop
F2<-function(recovery){
  aux_line      <- s_out[nrow(s_out),]
  age           = aux_line$age_y
  treatment     = aux_line$treatment
  episode       = aux_line$episode

  ## Transition probabilities ##
  # Help seeking & treatment

  p_help = if (aux_line$ltdm == 1){
    c(1-(reach_treatment*(1+ltdm_coordination)),(reach_treatment*(1+ltdm_coordination)))
  }else {c( 1-reach_treatment,reach_treatment)} #Better chances for help seeking within ltdm

  if (p_help[1]<=0){p_help = c(0,1)}else{NULL}

  if(aux_line$help_seeking == 0){
     aux_line$help_seeking = sample(c(0:1),1, replace = T, prob = p_help)
  } else {NULL} # Person starts looking for help ?

  if(aux_line$treatment_t == tlimit_treatment){aux_line$treatment=0; aux_line$help_seeking=0; aux_line$waiting=0}else{NULL}
  # If time limit treatment is reached, end treatment

  if(aux_line$help_seeking == 1 && aux_line$waiting == 0 && aux_line$treatment == 0 && aux_line$ltdm == 0) {
    aux_line$waiting =abs(round(rnorm(1, mean=delay_treatment, sd=1)))} else {NULL} # Start treatment

  if(aux_line$help_seeking == 1 && aux_line$waiting == 0 && aux_line$treatment == 0 && aux_line$ltdm == 1) {
    aux_line$waiting =abs(round(rnorm(1, mean=(delay_treatment*(1-ltdm_coordination)), sd=1)))} else {NULL} # Start treatment

  if(aux_line$help_seeking == 1 && aux_line$waiting <= 1 && aux_line$treatment_t < tlimit_treatment) {
    aux_line$treatment=1} else {NULL} # Start treatment

  if(aux_line$help_seeking == 1 && aux_line$waiting >= 1) {
    aux_line$waiting=aux_line$waiting-1} else {NULL} # Count waiting time backwards

  # Transition to healthy
  p_healthy = recovery
  if (treatment == 1){p_healthy = p_healthy*(1+effect_treatment)} else {NULL}  # Effect treatment
  h_state = sample(TS, 1, replace = T, prob = c (p_healthy,1-p_healthy))

  # Transition to death
  p_death = lookup_prob(age_tables, type = "mortality", age, gender)
  if (h_state == 2){p_death = p_death*lookup_prob(age_tables, type = "excess_factor", age, gender)}else {NULL}
  d_state = sample(DS, 1, replace = T, prob = c (1-p_death,p_death))

  # Transition
  transition_state = if(d_state==1){3}else{h_state}

  ## Decide if person receives ltdm
  if(transition_state == 1 && treatment == 1 && aux_line$ltdm == 0  && aux_line$chronic == 1){aux_line$ltdm =
    sample(c(0,1),1, replace = T, prob = c(1-reach_ltdm, reach_ltdm))} else {NULL}
  if(aux_line$ltdm == 1 && aux_line$ltdm_t == 0){aux_line$ltdm_t =
    tlimit_ltdm} else {NULL}

  ## Decide if person receives aftercare
  if(transition_state == 1 && treatment == 1 && aux_line$ltdm == 0){aux_line$aftercare =
    sample(c(0,1),1, replace = T, prob = c(1-reach_aftercare, reach_aftercare))} else {NULL}
  if(transition_state == 1 && aux_line$aftercare == 1){aux_line$aftercare_t =
    tlimit_aftercare} else {NULL}

  ## Save new state and prepare next loop
  if(transition_state != aux_line$state) {aux_line$transition=1} else {NULL} # Indicate state transition
  aux_line$state       = transition_state           # Transition
  aux_line$age_m       = aux_line$age_m+1           # Person ages for one month
  aux_line$age_y       = trunc(aux_line$age_m/12, 0) # Age in years

  if(transition_state == 2){ aux_line$time_depression  = aux_line$time_depression+1;
  aux_line$total_depression = aux_line$total_depression+1
  }else {aux_line$time_depression = 0} # Count time_depression

  if(aux_line$treatment == 1 && transition_state == 1) {aux_line$treatment = 0} else {NULL}
```

```
  if(transition_state   == 1) {aux_line$help_seeking = 0} else {NULL}

  if(transition_state   == 1) {aux_line$waiting = 0} else {NULL}
  if(aux_line$chronic   == 1 |aux_line$episode >= ltdm_en  |  aux_line$time_depression >= ltdm_ed) {aux_line$chronic=1} else {NULL}
  if(aux_line$chronic_24 == 0 && aux_line$time_depression > 24) {aux_line$chronic_24=1} else {NULL}
  if(aux_line$treatment == 1){aux_line$treatment_t = aux_line$treatment_t+1} else {aux_line$treatment_t = 0}

  if(aux_line$aftercare_t != tlimit_aftercare && aux_line$aftercare_t>=1){aux_line$aftercare_t = aux_line$aftercare_t-1} else {NULL}
  # Count time in aftercare backwards
  if(aux_line$aftercare_t==0){aux_line$aftercare = 0} else {NULL}
  # If time limit aftercare is reached, end aftercare

  if(aux_line$ltdm_t != tlimit_ltdm && aux_line$ltdm_t >=1) {aux_line$ltdm_t = aux_line$ltdm_t-1} else {NULL}
  if(aux_line$ltdm_t == 0){aux_line$ltdm = 0} else {NULL}

  s_out <- dplyr::bind_rows(s_out,aux_line) #, make.row.names=F)
 }

 IDX<-1              # Start with ID Nr. 1
 s_out<-createperson() # Create empty DF for loop
 Daten<-s_out          # Create empty DF for results
 gender<- s_out$gender # Load gender for first loop

 (for (i in 1:Num) {
  repeat {
   if(s_out[nrow(s_out),]$state == 1){
     s_out <- F1()
   } else if(s_out[nrow(s_out),]$state == 2) {
     s_out <-F2(recovery = recovery)
   } else {s_out<-head(s_out,-1);break}
  }

  ### Complete Data ###
  if(complete_data) {
   Daten <- dplyr::bind_rows(Daten, s_out)
  }else {    ### Result Data ###
   S_Line$ID<-s_out[nrow(s_out),]$ID
   S_Line$gender<-s_out[nrow(s_out),]$gender
   S_Line$age_y<-s_out[nrow(s_out),]$age_y
   S_Line$age_m<-s_out[nrow(s_out),]$age_m
   S_Line$episode<-s_out[nrow(s_out),]$episode
   S_Line$help_seeking<-base::max(s_out$help_seeking)
   S_Line$treatment<-base::sum(s_out$treatment)
   S_Line$total_depression<-s_out[nrow(s_out),]$total_depression
   S_Line$chronic<-s_out[nrow(s_out),]$chronic
   S_Line$persistent<-base::max(s_out$chronic_24)
   S_Line$aftercare<-base::sum(s_out$aftercare)
   S_Line$ltdm<-base::sum(s_out$ltdm)
   S_Line$s_death<-s_out[nrow(s_out),]$state
   if(S_Line$episode>0){S_Line$onset<-min(s_out[s_out$state==2 & s_out$episode ==1,"age_y"])}else{S_Line$onset<-0}
     S_Result <- dplyr::bind_rows(S_Result, S_Line)
   }

  IDX<-IDX+1
  s_out <- createperson(n_count)
  gender<- s_out$gender
  if (IDX %% 1e2 == 0){
   gc()} #free up memory after every 100 iterations
 })


 if(complete_data) {
  ### Whole Data Set ###
  filename <- paste0(pars$file, "_C.csv")
  write.csv2(Daten, file = filename)
 } else {
  ### Summary ###
  filename2 <- paste0(pars$file, "_R.csv")
  write.csv2(S_Result[-1,], file = filename2)
 }
 gc()
}
```

```
# 3 Parallel computing on MAC ---------------------------------------------------

# Specify iterations (N) for each simulation
#Num<-100

# Prepare for parallel computing
#cffork<-parallel::detectCores()-1     # one cluster less than cores on the computer to prevent overload
#cl<-parallel::makeForkCluster(cffork) # creates a set of copies of R running in parallel
#doParallel::registerDoParallel(cl)    # register cores

# Apply function in parallel
#system.time(foreach(i=params_list, .options.RNG=seed) %dorng% loop_f(i, Num = Num, complete_data = F))

# Stop cluster at the end of script#
#parallel::stopCluster(cl)

# 4 Parallel computing on HPC ---------------------------------------------------
# Specify iterations (N) for each simulation
#Num<-100

# Print out number of processes/tasks and nodes
#slurm_tasks <- as.numeric(Sys.getenv("SLURM_NTASKS"))
#print(slurm_tasks)
#slurm_nodes <- as.numeric(Sys.getenv("SLURM_NNODES"))
#print(slurm_nodes)

# Register Cores
#doParallel::registerDoParallel(cores = slurm_tasks)

#Apply function in parallel
#system.time(foreach(i=params_list, .options.RNG=seed) %dorng% loop_f(i, Num = Num, complete_data = F))

# Stop cluster at the end of script
#stopImplicitCluster()

# 5 Parallel Computing on Windows -----------------------------------------------

# Specify iterations (N) for each simulation
#Num<-100

#cffork<-parallel::detectCores()-1 # one cluster less than cores on the computer to prevent overload
#cl <- makeCluster(cffork, type="SOCK")
#doParallel::registerDoParallel(cl) #register cores

#clusterExport(cl, c("loop_f", "params_list", "S_Line", "S_Result", "relapse",
#                    "age_tables", "Num"))

# Apply function in parallel
#system.time(foreach(i=params_list, .options.RNG=seed) %dorng% loop_f(i, Num = Num, complete_data = F))

# Stop cluster at the end of script
#parallel::stopCluster(cl)
```

Table S1: Parameter settings for model variation (set01001.csv).

file	effect_ prevention	reach_ prevention	effect_ treatment	reach_ treatment	tlimit_ treatment	effect_ aftercare	reach_ aftercare	tlimit_ aftercare	effect_ ltdm	reach_ ltdm	tlimit_ ltdm	ltdm_ coordination
001_base	0,21	0,05	0,62	0,33	24	0,36	0,05	12	0,31	0,05	24	0,5
001_nhc	0,21	0	0,62	0	24	0,36	0	12	0,31	0	24	0,5
001_pe_46	0,46	0,05	0,62	0,33	24	0,36	0,05	12	0,31	0,05	24	0,5
001_pr_3	0,21	0,3	0,62	0,33	24	0,36	0,05	12	0,31	0,05	24	0,5
001_te_87	0,21	0,05	0,87	0,33	24	0,36	0,05	12	0,31	0,05	24	0,5
001_tr_58	0,21	0,05	0,62	0,58	24	0,36	0,05	12	0,31	0,05	24	0,5
001_ae_61	0,21	0,05	0,62	0,33	24	0,61	0,05	12	0,31	0,05	24	0,5
001_ar_3	0,21	0,05	0,62	0,33	24	0,36	0,3	12	0,31	0,05	24	0,5
001_me_56	0,21	0,05	0,62	0,33	24	0,36	0,05	12	0,56	0,05	24	0,5
001_mr_3	0,21	0,05	0,62	0,33	24	0,36	0,05	12	0,31	0,3	24	0,5
001_pe_1	1	0,05	0,62	0,33	24	0,36	0,05	12	0,31	0,05	24	0,5
001_pr_1	0,21	1	0,62	0,33	24	0,36	0,05	12	0,31	0,05	24	0,5
001_te_1	0,21	0,05	1	0,33	24	0,36	0,05	12	0,31	0,05	24	0,5
001_tr_1	0,21	0,05	0,62	1	24	0,36	0,05	12	0,31	0,05	24	0,5
001_ae_1	0,21	0,05	0,62	0,33	24	1	0,05	12	0,31	0,05	24	0,5
001_ar_1	0,21	0,05	0,62	0,33	24	0,36	1	12	0,31	0,05	24	0,5
001_me_1	0,21	0,05	0,62	0,33	24	0,36	0,05	12	1	0,05	24	0,5
001_mr_1	0,21	0,05	0,62	0,33	24	0,36	0,05	12	0,31	1	24	0,5

Note: To run the script, the table must be saved in csv format in the working directory and named “set01001.csv”.

Table S2: Incidence-, mortality-, and excess mortality-rates (age_tables.csv).

age	incidence_m	incidence_f	mortality_m	mortality_f	excess_factor_m	excess_factor_f
0	0,0000319	5,4198E-05	0,00029047	0,00024382	1,48	1,33
1	0,0000319	5,4198E-05	2,23E-05	1,95E-05	1,48	1,33
2	0,0000319	5,4198E-05	1,24E-05	1,05E-05	1,48	1,33
3	0,0000319	5,4198E-05	9,93E-06	7,87E-06	1,48	1,33
4	0,0000319	5,4198E-05	8,71E-06	8,51E-06	1,48	1,33
5	0,0000319	5,4198E-05	7,99E-06	7,28E-06	1,48	1,33
6	0,0000319	5,4198E-05	7,41E-06	7,05E-06	1,48	1,33
7	6,38E-05	1,08E-04	7,14E-06	4,71E-06	1,48	1,33
8	6,38E-05	1,08E-04	6,15E-06	5,48E-06	1,48	1,33
9	6,38E-05	1,08E-04	6,69E-06	4,12E-06	1,48	1,33
10	6,38E-05	1,08E-04	6,32E-06	4,59E-06	1,48	1,33
11	6,38E-05	1,08E-04	6,11E-06	6,94E-06	1,48	1,33
12	6,38E-05	1,08E-04	6,84E-06	6,00E-06	1,48	1,33
13	6,38E-05	1,08E-04	7,52E-06	7,50E-06	1,48	1,33
14	6,38E-05	1,08E-04	1,10E-05	7,72E-06	1,48	1,33
15	6,38E-05	1,08E-04	1,36E-05	9,97E-06	1,48	1,33
16	6,38E-05	1,08E-04	1,88E-05	1,21E-05	1,48	1,33
17	6,38E-05	1,08E-04	2,20E-05	1,05E-05	1,48	1,33
18	6,38E-05	1,08E-04	3,25E-05	1,52E-05	4,48	3,92
19	6,38E-05	1,08E-04	3,32E-05	1,40E-05	4,48	3,92
20	6,38E-05	1,08E-04	3,50E-05	1,50E-05	4,48	3,92
21	6,38E-05	1,08E-04	3,37E-05	1,44E-05	4,48	3,92
22	6,38E-05	1,08E-04	3,50E-05	1,39E-05	4,48	3,92
23	6,38E-05	1,08E-04	3,33E-05	1,36E-05	4,48	3,92
24	6,38E-05	1,08E-04	3,87E-05	1,54E-05	4,48	3,92
25	8,30E-05	0,00014475	3,65E-05	1,58E-05	2,6	4,44
26	8,30E-05	0,00014475	3,71E-05	1,47E-05	2,6	4,44
27	8,30E-05	0,00014475	3,70E-05	1,77E-05	2,6	4,44
28	8,30E-05	0,00014475	4,09E-05	1,93E-05	2,6	4,44
29	8,30E-05	0,00014475	4,15E-05	2,17E-05	2,6	4,44
30	8,94E-05	0,00015689	4,50E-05	2,54E-05	2,6	4,44
31	8,94E-05	0,00015689	5,04E-05	2,68E-05	2,6	4,44
32	8,94E-05	0,00015689	5,21E-05	2,80E-05	2,6	4,44
33	8,94E-05	0,00015689	6,13E-05	2,93E-05	2,6	4,44
34	8,94E-05	0,00015689	5,98E-05	3,36E-05	2,6	4,44
35	0,00010219	0,00016904	6,30E-05	3,71E-05	2,47	2,45
36	0,00010219	0,00016904	7,28E-05	3,88E-05	2,47	2,45
37	0,00010219	0,00016904	8,18E-05	4,16E-05	2,47	2,45
38	0,00010219	0,00016904	8,50E-05	4,78E-05	2,47	2,45
39	0,00010219	0,00016904	9,24E-05	5,31E-05	2,47	2,45
40	0,00011502	0,00016296	9,91E-05	5,40E-05	2,47	2,45
41	0,00011502	0,00016296	0,00011658	5,91E-05	2,47	2,45
42	0,00011502	0,00016296	0,00011631	6,56E-05	2,47	2,45
43	0,00011502	0,00016296	0,00012969	7,40E-05	2,47	2,45
44	0,00011502	0,00016296	0,00014305	7,84E-05	2,47	2,45
45	0,00012144	0,00016904	0,00015993	9,18E-05	1,92	1,81
46	0,00012144	0,00016904	0,0001774	0,00010102	1,92	1,81
47	0,00012144	0,00016904	0,00019754	0,00011065	1,92	1,81
48	0,00012144	0,00016904	0,0002183	0,00012536	1,92	1,81
49	0,00012144	0,00016904	0,00024091	0,00014169	1,92	1,81
50	0,00012786	0,0001812	0,00026809	0,00015427	1,92	1,81
51	0,00012786	0,0001812	0,00030006	0,00017716	1,92	1,81
52	0,00012786	0,0001812	0,00033747	0,00018955	1,92	1,81
53	0,00012786	0,0001812	0,00038071	0,00021216	1,92	1,81
54	0,00012786	0,0001812	0,00042852	0,0002346	1,92	1,81
55	0,00014072	0,0001812	0,00047786	0,00026203	1,26	1,2
56	0,00014072	0,0001812	0,00053475	0,00029307	1,26	1,2
57	0,00014072	0,0001812	0,00059933	0,00032133	1,26	1,2
58	0,00014072	0,0001812	0,00065182	0,00035102	1,26	1,2
59	0,00014072	0,0001812	0,00073471	0,00038602	1,26	1,2
60	0,00012786	0,00015689	0,00080366	0,00043061	1,15	1,29
61	0,00012786	0,00015689	0,00089258	0,00047651	1,15	1,29
62	0,00012786	0,00015689	0,00098758	0,00051287	1,15	1,29
63	0,00012786	0,00015689	0,00108245	0,00056506	1,15	1,29
64	0,00012786	0,00015689	0,001183	0,00061932	1,15	1,29
65	0,00011502	0,00015081	0,00128028	0,00066426	1,26	1,61
66	0,00011502	0,00015081	0,00139867	0,00073222	1,26	1,61
67	0,00011502	0,00015081	0,00150732	0,00078882	1,26	1,61
68	0,00011502	0,00015081	0,0016325	0,0008624	1,26	1,61
69	0,00011502	0,00015081	0,00174411	0,00095791	1,26	1,61
70	0,00012786	0,00016904	0,00190656	0,00106602	1,6	1,57
71	0,00012786	0,00016904	0,00204936	0,00113994	1,6	1,57
72	0,00012786	0,00016904	0,00222523	0,00126371	1,6	1,57
73	0,00012786	0,00016904	0,00240212	0,0013864	1,6	1,57
74	0,00012786	0,00016904	0,00264011	0,00152413	1,6	1,57
75	0,00015358	0,00019338	0,00287035	0,00167268	1,58	1,43
76	0,00015358	0,00019338	0,00311355	0,00180536	1,58	1,43
77	0,00015358	0,00019338	0,00343882	0,00201229	1,58	1,43
78	0,00015358	0,00019338	0,00379182	0,00227443	1,58	1,43
79	0,00015358	0,00019338	0,00425894	0,00262389	1,58	1,43
80	0,00019227	0,00022998	0,00474145	0,00300846	1,54	1,32
81	0,00019227	0,00022998	0,00536449	0,00349801	1,54	1,32
82	0,00019227	0,00022998	0,00611233	0,00413084	1,54	1,32
83	0,00019227	0,00022998	0,00687046	0,00475664	1,54	1,32
84	0,00019227	0,00022998	0,00789406	0,00553676	1,54	1,32
85	0,00020519	0,00024221	0,00891981	0,00640276	1,31	1,29
86	0,00020519	0,00024221	0,01022119	0,00747902	1,31	1,29
87	0,00020519	0,00024221	0,01145787	0,00865171	1,31	1,29
88	0,00020519	0,00024221	0,01320037	0,01000541	1,31	1,29
89	0,00020519	0,00024221	0,0148179	0,01142848	1,31	1,29
90	0,00019873	0,00024221	0,01661438	0,01318546	1,44	1,17

91	0,00019873	0,00024221	0,01819966	0,01500542	1,44	1,17
92	0,00019873	0,00024221	0,02089829	0,01720799	1,44	1,17
93	0,00019873	0,00024221	0,02289821	0,01937773	1,44	1,17
94	0,00019873	0,00024221	0,02560873	0,02138016	1,44	1,17
95	0,00019873	0,0001812	0,02787805	0,02411727	1,04	1,14
96	0,00019873	0,0001812	0,0310275	0,02679583	1,04	1,14
97	0,00019873	0,0001812	0,03425927	0,02860936	1,04	1,14
98	0,00019873	0,0001812	0,03630609	0,03133363	1,04	1,14
99	0,00019873	0,0001812	0,03926449	0,03413356	1,04	1,14
100	0,00019873	0,0001812	0,04223745	0,03698255	1,04	1,14
101	0,00019873	0,0001812	0,04223745	0,03983154	1,04	1,14
102	0,00019873	0,0001812	0,04521041	0,04268053	1,04	1,14
103	0,00019873	0,0001812	0,04818337	0,04552952	1,04	1,14
104	0,00019873	0,0001812	0,05115633	0,04837851	1,04	1,14
105	0,00019873	0,0001812	0,05412929	0,0512275	1,04	1,14
106	0,00019873	0,0001812	0,05710225	0,05407649	1,04	1,14
107	0,00019873	0,0001812	0,06007521	0,05692548	1,04	1,14
108	0,00019873	0,0001812	0,06304817	0,05977447	1,04	1,14
109	0,00019873	0,0001812	0,06602113	0,06262346	1,04	1,14
110	0,00019873	0,0001812	0,06899409	0,06547245	1,04	1,14
111	0,00019873	0,0001812	0,07196705	0,06832144	1,04	1,14
112	0,00019873	0,0001812	0,07494001	0,07117043	1,04	1,14
113	0,00019873	0,0001812	0,07791297	0,07401942	1,04	1,14
114	0,00019873	0,0001812	0,08088593	0,07686841	1,04	1,14
115	0,00019873	0,0001812	0,08385889	0,0797174	1,04	1,14
116	0,00019873	0,0001812	0,08683185	0,08256639	1,04	1,14
117	0,00019873	0,0001812	0,08980481	0,08541538	1,04	1,14
118	0,00019873	0,0001812	0,09277777	0,08826437	1,04	1,14
119	0,00019873	0,0001812	0,09575073	0,09111336	1,04	1,14
120	0,00019873	0,0001812	0,09872369	0,09396235	1,04	1,14
121	0,00019873	0,0001812	0,10169665	0,09681134	1,04	1,14
122	0,00019873	0,0001812	0,10466961	0,09966033	1,04	1,14
123	0,00019873	0,0001812	0,10764257	0,10250932	1,04	1,14
124	0,00019873	0,0001812	0,11061553	0,10535831	1,04	1,14
125	0,00019873	0,0001812	0,11358849	0,1082073	1,04	1,14
126	0,00019873	0,0001812	0,11656145	0,11105629	1,04	1,14
127	0,00019873	0,0001812	0,11953441	0,11390528	1,04	1,14
128	0,00019873	0,0001812	0,12250737	0,11675427	1,04	1,14
129	0,00019873	0,0001812	0,12548033	0,11960326	1,04	1,14
130	0,00019873	0,0001812	0,12845329	0,12245225	1,04	1,14
131	0,00019873	0,0001812	0,13142625	0,12530124	1,04	1,14
132	0,00019873	0,0001812	0,13439921	0,12815023	1,04	1,14
133	0,00019873	0,0001812	0,13737217	0,13099922	1,04	1,14
134	0,00019873	0,0001812	0,14034513	0,13384821	1,04	1,14
135	0,00019873	0,0001812	0,14331809	0,1366972	1,04	1,14
136	0,00019873	0,0001812	0,14629105	0,13954619	1,04	1,14
137	0,00019873	0,0001812	0,14926401	0,14239518	1,04	1,14
138	0,00019873	0,0001812	0,15223697	0,14524417	1,04	1,14
139	0,00019873	0,0001812	0,15520993	0,14809316	1,04	1,14
140	0,00019873	0,0001812	0,15818289	0,15094215	1,04	1,14
141	0,00019873	0,0001812	0,16115585	0,15379114	1,04	1,14
142	0,00019873	0,0001812	0,16412881	0,15664013	1,04	1,14
143	0,00019873	0,0001812	0,16710177	0,15948912	1,04	1,14
144	0,00019873	0,0001812	0,17007473	0,16233811	1,04	1,14
145	0,00019873	0,0001812	0,17304769	0,1651871	1,04	1,14
146	0,00019873	0,0001812	0,17602065	0,16803609	1,04	1,14
147	0,00019873	0,0001812	0,17899361	0,17088508	1,04	1,14
148	0,00019873	0,0001812	0,18196657	0,17373407	1,04	1,14
149	0,00019873	0,0001812	0,18493953	0,17658306	1,04	1,14
150	0,00019873	0,0001812	0,18791249	0,17943205	1,04	1,14

Note: To run the script, the table must be saved in csv format in the working directory and named “age_tables.csv”.

Table S3: Relapse probability for monthly transition from healthy to depressed (relapse.csv).

time	E_1	E_2	E_3	E_4	E_5	E_6
0	0	0	0	0	0	0
1	0,02368842	0,02747857	0,03187514	0,03697517	0,04289119	0,04289119
2	0,02368842	0,02747857	0,03187514	0,03697517	0,04289119	0,04289119
3	0,02368842	0,02747857	0,03187514	0,03697517	0,04289119	0,04289119
4	0,02368842	0,02747857	0,03187514	0,03697517	0,04289119	0,04289119
5	0,02368842	0,02747857	0,03187514	0,03697517	0,04289119	0,04289119
6	0,02368842	0,02747857	0,03187514	0,03697517	0,04289119	0,04289119
7	0,02368842	0,02747857	0,03187514	0,03697517	0,04289119	0,04289119
8	0,02368842	0,02747857	0,03187514	0,03697517	0,04289119	0,04289119
9	0,02368842	0,02747857	0,03187514	0,03697517	0,04289119	0,04289119
10	0,02368842	0,02747857	0,03187514	0,03697517	0,04289119	0,04289119
11	0,02368842	0,02747857	0,03187514	0,03697517	0,04289119	0,04289119
12	0,02368842	0,02747857	0,03187514	0,03697517	0,04289119	0,04289119
13	0,01540754	0,01787274	0,02073238	0,02404956	0,02789749	0,02789749
14	0,01540754	0,01787274	0,02073238	0,02404956	0,02789749	0,02789749
15	0,01540754	0,01787274	0,02073238	0,02404956	0,02789749	0,02789749
16	0,01540754	0,01787274	0,02073238	0,02404956	0,02789749	0,02789749
17	0,01540754	0,01787274	0,02073238	0,02404956	0,02789749	0,02789749
18	0,01540754	0,01787274	0,02073238	0,02404956	0,02789749	0,02789749
19	0,01540754	0,01787274	0,02073238	0,02404956	0,02789749	0,02789749
20	0,01540754	0,01787274	0,02073238	0,02404956	0,02789749	0,02789749
21	0,01540754	0,01787274	0,02073238	0,02404956	0,02789749	0,02789749
22	0,01540754	0,01787274	0,02073238	0,02404956	0,02789749	0,02789749
23	0,01540754	0,01787274	0,02073238	0,02404956	0,02789749	0,02789749
24	0,01540754	0,01787274	0,02073238	0,02404956	0,02789749	0,02789749
25	0,00549736	0,00637694	0,00739725	0,00858081	0,00995374	0,00995374
26	0,00549736	0,00637694	0,00739725	0,00858081	0,00995374	0,00995374
27	0,00549736	0,00637694	0,00739725	0,00858081	0,00995374	0,00995374
28	0,00549736	0,00637694	0,00739725	0,00858081	0,00995374	0,00995374
29	0,00549736	0,00637694	0,00739725	0,00858081	0,00995374	0,00995374
30	0,00549736	0,00637694	0,00739725	0,00858081	0,00995374	0,00995374
31	0,00549736	0,00637694	0,00739725	0,00858081	0,00995374	0,00995374
32	0,00549736	0,00637694	0,00739725	0,00858081	0,00995374	0,00995374
33	0,00549736	0,00637694	0,00739725	0,00858081	0,00995374	0,00995374
34	0,00549736	0,00637694	0,00739725	0,00858081	0,00995374	0,00995374
35	0,00549736	0,00637694	0,00739725	0,00858081	0,00995374	0,00995374
36	0,00549736	0,00637694	0,00739725	0,00858081	0,00995374	0,00995374
37	0,00549736	0,00637694	0,00739725	0,00858081	0,00995374	0,00995374
38	0,00549736	0,00637694	0,00739725	0,00858081	0,00995374	0,00995374
39	0,00549736	0,00637694	0,00739725	0,00858081	0,00995374	0,00995374
40	0,00549736	0,00637694	0,00739725	0,00858081	0,00995374	0,00995374
41	0,00549736	0,00637694	0,00739725	0,00858081	0,00995374	0,00995374
42	0,00549736	0,00637694	0,00739725	0,00858081	0,00995374	0,00995374
43	0,00549736	0,00637694	0,00739725	0,00858081	0,00995374	0,00995374
44	0,00549736	0,00637694	0,00739725	0,00858081	0,00995374	0,00995374
45	0,00549736	0,00637694	0,00739725	0,00858081	0,00995374	0,00995374
46	0,00549736	0,00637694	0,00739725	0,00858081	0,00995374	0,00995374
47	0,00549736	0,00637694	0,00739725	0,00858081	0,00995374	0,00995374
48	0,00549736	0,00637694	0,00739725	0,00858081	0,00995374	0,00995374
49	0,00549736	0,00637694	0,00739725	0,00858081	0,00995374	0,00995374
50	0,00549736	0,00637694	0,00739725	0,00858081	0,00995374	0,00995374
51	0,00549736	0,00637694	0,00739725	0,00858081	0,00995374	0,00995374
52	0,00549736	0,00637694	0,00739725	0,00858081	0,00995374	0,00995374
53	0,00549736	0,00637694	0,00739725	0,00858081	0,00995374	0,00995374
54	0,00549736	0,00637694	0,00739725	0,00858081	0,00995374	0,00995374
55	0,00549736	0,00637694	0,00739725	0,00858081	0,00995374	0,00995374
56	0,00549736	0,00637694	0,00739725	0,00858081	0,00995374	0,00995374
57	0,00549736	0,00637694	0,00739725	0,00858081	0,00995374	0,00995374
58	0,00549736	0,00637694	0,00739725	0,00858081	0,00995374	0,00995374
59	0,00549736	0,00637694	0,00739725	0,00858081	0,00995374	0,00995374
60	0,00549736	0,00637694	0,00739725	0,00858081	0,00995374	0,00995374
61	0,00175447	0,00203518	0,00236081	0,00273854	0,00317671	0,00317671
62	0,00175447	0,00203518	0,00236081	0,00273854	0,00317671	0,00317671
63	0,00175447	0,00203518	0,00236081	0,00273854	0,00317671	0,00317671
64	0,00175447	0,00203518	0,00236081	0,00273854	0,00317671	0,00317671
65	0,00175447	0,00203518	0,00236081	0,00273854	0,00317671	0,00317671
66	0,00175447	0,00203518	0,00236081	0,00273854	0,00317671	0,00317671
67	0,00175447	0,00203518	0,00236081	0,00273854	0,00317671	0,00317671
68	0,00175447	0,00203518	0,00236081	0,00273854	0,00317671	0,00317671
69	0,00175447	0,00203518	0,00236081	0,00273854	0,00317671	0,00317671
70	0,00175447	0,00203518	0,00236081	0,00273854	0,00317671	0,00317671
71	0,00175447	0,00203518	0,00236081	0,00273854	0,00317671	0,00317671
72	0,00175447	0,00203518	0,00236081	0,00273854	0,00317671	0,00317671
73	0,00175447	0,00203518	0,00236081	0,00273854	0,00317671	0,00317671
74	0,00175447	0,00203518	0,00236081	0,00273854	0,00317671	0,00317671
75	0,00175447	0,00203518	0,00236081	0,00273854	0,00317671	0,00317671
76	0,00175447	0,00203518	0,00236081	0,00273854	0,00317671	0,00317671
77	0,00175447	0,00203518	0,00236081	0,00273854	0,00317671	0,00317671
78	0,00175447	0,00203518	0,00236081	0,00273854	0,00317671	0,00317671
79	0,00175447	0,00203518	0,00236081	0,00273854	0,00317671	0,00317671
80	0,00175447	0,00203518	0,00236081	0,00273854	0,00317671	0,00317671
81	0,00175447	0,00203518	0,00236081	0,00273854	0,00317671	0,00317671
82	0,00175447	0,00203518	0,00236081	0,00273854	0,00317671	0,00317671
83	0,00175447	0,00203518	0,00236081	0,00273854	0,00317671	0,00317671
84	0,00175447	0,00203518	0,00236081	0,00273854	0,00317671	0,00317671
85	0,00175447	0,00203518	0,00236081	0,00273854	0,00317671	0,00317671
86	0,00175447	0,00203518	0,00236081	0,00273854	0,00317671	0,00317671
87	0,00175447	0,00203518	0,00236081	0,00273854	0,00317671	0,00317671
88	0,00175447	0,00203518	0,00236081	0,00273854	0,00317671	0,00317671
89	0,00175447	0,00203518	0,00236081	0,00273854	0,00317671	0,00317671
90	0,00175447	0,00203518	0,00236081	0,00273854	0,00317671	0,00317671

91	0,00175447	0,00203518	0,00236081	0,00273854	0,00317671	0,00317671
92	0,00175447	0,00203518	0,00236081	0,00273854	0,00317671	0,00317671
93	0,00175447	0,00203518	0,00236081	0,00273854	0,00317671	0,00317671
94	0,00175447	0,00203518	0,00236081	0,00273854	0,00317671	0,00317671
95	0,00175447	0,00203518	0,00236081	0,00273854	0,00317671	0,00317671
96	0,00175447	0,00203518	0,00236081	0,00273854	0,00317671	0,00317671
97	0,00175447	0,00203518	0,00236081	0,00273854	0,00317671	0,00317671
98	0,00175447	0,00203518	0,00236081	0,00273854	0,00317671	0,00317671
99	0,00175447	0,00203518	0,00236081	0,00273854	0,00317671	0,00317671
100	0,00175447	0,00203518	0,00236081	0,00273854	0,00317671	0,00317671
101	0,00175447	0,00203518	0,00236081	0,00273854	0,00317671	0,00317671
102	0,00175447	0,00203518	0,00236081	0,00273854	0,00317671	0,00317671
103	0,00175447	0,00203518	0,00236081	0,00273854	0,00317671	0,00317671
104	0,00175447	0,00203518	0,00236081	0,00273854	0,00317671	0,00317671
105	0,00175447	0,00203518	0,00236081	0,00273854	0,00317671	0,00317671
106	0,00175447	0,00203518	0,00236081	0,00273854	0,00317671	0,00317671
107	0,00175447	0,00203518	0,00236081	0,00273854	0,00317671	0,00317671
108	0,00175447	0,00203518	0,00236081	0,00273854	0,00317671	0,00317671
109	0,00175447	0,00203518	0,00236081	0,00273854	0,00317671	0,00317671
110	0,00175447	0,00203518	0,00236081	0,00273854	0,00317671	0,00317671
111	0,00175447	0,00203518	0,00236081	0,00273854	0,00317671	0,00317671
112	0,00175447	0,00203518	0,00236081	0,00273854	0,00317671	0,00317671
113	0,00175447	0,00203518	0,00236081	0,00273854	0,00317671	0,00317671
114	0,00175447	0,00203518	0,00236081	0,00273854	0,00317671	0,00317671
115	0,00175447	0,00203518	0,00236081	0,00273854	0,00317671	0,00317671
116	0,00175447	0,00203518	0,00236081	0,00273854	0,00317671	0,00317671
117	0,00175447	0,00203518	0,00236081	0,00273854	0,00317671	0,00317671
118	0,00175447	0,00203518	0,00236081	0,00273854	0,00317671	0,00317671
119	0,00175447	0,00203518	0,00236081	0,00273854	0,00317671	0,00317671
120	0,00175447	0,00203518	0,00236081	0,00273854	0,00317671	0,00317671
121	0,00042735	0,00049573	0,00057505	0,00066705	0,00077378	0,00089759
122	0,00042735	0,00049573	0,00057505	0,00066705	0,00077378	0,00089759
123	0,00042735	0,00049573	0,00057505	0,00066705	0,00077378	0,00089759
124	0,00042735	0,00049573	0,00057505	0,00066705	0,00077378	0,00089759
125	0,00042735	0,00049573	0,00057505	0,00066705	0,00077378	0,00089759
126	0,00042735	0,00049573	0,00057505	0,00066705	0,00077378	0,00089759
127	0,00042735	0,00049573	0,00057505	0,00066705	0,00077378	0,00089759
128	0,00042735	0,00049573	0,00057505	0,00066705	0,00077378	0,00089759
129	0,00042735	0,00049573	0,00057505	0,00066705	0,00077378	0,00089759
130	0,00042735	0,00049573	0,00057505	0,00066705	0,00077378	0,00089759
131	0,00042735	0,00049573	0,00057505	0,00066705	0,00077378	0,00089759
132	0,00042735	0,00049573	0,00057505	0,00066705	0,00077378	0,00089759
133	0,00042735	0,00049573	0,00057505	0,00066705	0,00077378	0,00089759
134	0,00042735	0,00049573	0,00057505	0,00066705	0,00077378	0,00089759
135	0,00042735	0,00049573	0,00057505	0,00066705	0,00077378	0,00089759
136	0,00042735	0,00049573	0,00057505	0,00066705	0,00077378	0,00089759
137	0,00042735	0,00049573	0,00057505	0,00066705	0,00077378	0,00089759
138	0,00042735	0,00049573	0,00057505	0,00066705	0,00077378	0,00089759
139	0,00042735	0,00049573	0,00057505	0,00066705	0,00077378	0,00089759
140	0,00042735	0,00049573	0,00057505	0,00066705	0,00077378	0,00089759
141	0,00042735	0,00049573	0,00057505	0,00066705	0,00077378	0,00089759
142	0,00042735	0,00049573	0,00057505	0,00066705	0,00077378	0,00089759
143	0,00042735	0,00049573	0,00057505	0,00066705	0,00077378	0,00089759
144	0,00042735	0,00049573	0,00057505	0,00066705	0,00077378	0,00089759
145	0,00042735	0,00049573	0,00057505	0,00066705	0,00077378	0,00089759
146	0,00042735	0,00049573	0,00057505	0,00066705	0,00077378	0,00089759
147	0,00042735	0,00049573	0,00057505	0,00066705	0,00077378	0,00089759
148	0,00042735	0,00049573	0,00057505	0,00066705	0,00077378	0,00089759
149	0,00042735	0,00049573	0,00057505	0,00066705	0,00077378	0,00089759
150	0,00042735	0,00049573	0,00057505	0,00066705	0,00077378	0,00089759
151	0,00042735	0,00049573	0,00057505	0,00066705	0,00077378	0,00089759
152	0,00042735	0,00049573	0,00057505	0,00066705	0,00077378	0,00089759
153	0,00042735	0,00049573	0,00057505	0,00066705	0,00077378	0,00089759
154	0,00042735	0,00049573	0,00057505	0,00066705	0,00077378	0,00089759
155	0,00042735	0,00049573	0,00057505	0,00066705	0,00077378	0,00089759
156	0,00042735	0,00049573	0,00057505	0,00066705	0,00077378	0,00089759
157	0,00042735	0,00049573	0,00057505	0,00066705	0,00077378	0,00089759
158	0,00042735	0,00049573	0,00057505	0,00066705	0,00077378	0,00089759
159	0,00042735	0,00049573	0,00057505	0,00066705	0,00077378	0,00089759
160	0,00042735	0,00049573	0,00057505	0,00066705	0,00077378	0,00089759
161	0,00042735	0,00049573	0,00057505	0,00066705	0,00077378	0,00089759
162	0,00042735	0,00049573	0,00057505	0,00066705	0,00077378	0,00089759
163	0,00042735	0,00049573	0,00057505	0,00066705	0,00077378	0,00089759
164	0,00042735	0,00049573	0,00057505	0,00066705	0,00077378	0,00089759
165	0,00042735	0,00049573	0,00057505	0,00066705	0,00077378	0,00089759
166	0,00042735	0,00049573	0,00057505	0,00066705	0,00077378	0,00089759
167	0,00042735	0,00049573	0,00057505	0,00066705	0,00077378	0,00089759
168	0,00042735	0,00049573	0,00057505	0,00066705	0,00077378	0,00089759
169	0,00042735	0,00049573	0,00057505	0,00066705	0,00077378	0,00089759
170	0,00042735	0,00049573	0,00057505	0,00066705	0,00077378	0,00089759
171	0,00042735	0,00049573	0,00057505	0,00066705	0,00077378	0,00089759
172	0,00042735	0,00049573	0,00057505	0,00066705	0,00077378	0,00089759
173	0,00042735	0,00049573	0,00057505	0,00066705	0,00077378	0,00089759
174	0,00042735	0,00049573	0,00057505	0,00066705	0,00077378	0,00089759
175	0,00042735	0,00049573	0,00057505	0,00066705	0,00077378	0,00089759
176	0,00042735	0,00049573	0,00057505	0,00066705	0,00077378	0,00089759
177	0,00042735	0,00049573	0,00057505	0,00066705	0,00077378	0,00089759
178	0,00042735	0,00049573	0,00057505	0,00066705	0,00077378	0,00089759
179	0,00042735	0,00049573	0,00057505	0,00066705	0,00077378	0,00089759
180	0,00042735	0,00049573	0,00057505	0,00066705	0,00077378	0,00089759
181	0,00042735	0,00049573	0,00057505	0,00066705	0,00077378	0,00089759
182	0,00042735	0,00049573	0,00057505	0,00066705	0,00077378	0,00089759
183	0,00042735	0,00049573	0,00057505	0,00066705	0,00077378	0,00089759
184	0,00042735	0,00049573	0,00057505	0,00066705	0,00077378	0,00089759
185	0,00042735	0,00049573	0,00057505	0,00066705	0,00077378	0,00089759
186	0,00042735	0,00049573	0,00057505	0,00066705	0,00077378	0,00089759

187	0,00042735	0,00049573	0,00057505	0,00066705	0,00077378	0,00089759
188	0,00042735	0,00049573	0,00057505	0,00066705	0,00077378	0,00089759
189	0,00042735	0,00049573	0,00057505	0,00066705	0,00077378	0,00089759
190	0,00042735	0,00049573	0,00057505	0,00066705	0,00077378	0,00089759
191	0,00042735	0,00049573	0,00057505	0,00066705	0,00077378	0,00089759
192	0,00042735	0,00049573	0,00057505	0,00066705	0,00077378	0,00089759
193	0,00042735	0,00049573	0,00057505	0,00066705	0,00077378	0,00089759
194	0,00042735	0,00049573	0,00057505	0,00066705	0,00077378	0,00089759
195	0,00042735	0,00049573	0,00057505	0,00066705	0,00077378	0,00089759
196	0,00042735	0,00049573	0,00057505	0,00066705	0,00077378	0,00089759
197	0,00042735	0,00049573	0,00057505	0,00066705	0,00077378	0,00089759
198	0,00042735	0,00049573	0,00057505	0,00066705	0,00077378	0,00089759
199	0,00042735	0,00049573	0,00057505	0,00066705	0,00077378	0,00089759
200	0,00042735	0,00049573	0,00057505	0,00066705	0,00077378	0,00089759
201	0,00042735	0,00049573	0,00057505	0,00066705	0,00077378	0,00089759
202	0,00042735	0,00049573	0,00057505	0,00066705	0,00077378	0,00089759
203	0,00042735	0,00049573	0,00057505	0,00066705	0,00077378	0,00089759
204	0,00042735	0,00049573	0,00057505	0,00066705	0,00077378	0,00089759
205	0,00042735	0,00049573	0,00057505	0,00066705	0,00077378	0,00089759
206	0,00042735	0,00049573	0,00057505	0,00066705	0,00077378	0,00089759
207	0,00042735	0,00049573	0,00057505	0,00066705	0,00077378	0,00089759
208	0,00042735	0,00049573	0,00057505	0,00066705	0,00077378	0,00089759
209	0,00042735	0,00049573	0,00057505	0,00066705	0,00077378	0,00089759
210	0,00042735	0,00049573	0,00057505	0,00066705	0,00077378	0,00089759
211	0,00042735	0,00049573	0,00057505	0,00066705	0,00077378	0,00089759
212	0,00042735	0,00049573	0,00057505	0,00066705	0,00077378	0,00089759
213	0,00042735	0,00049573	0,00057505	0,00066705	0,00077378	0,00089759
214	0,00042735	0,00049573	0,00057505	0,00066705	0,00077378	0,00089759
215	0,00042735	0,00049573	0,00057505	0,00066705	0,00077378	0,00089759
216	0,00042735	0,00049573	0,00057505	0,00066705	0,00077378	0,00089759
217	0,00042735	0,00049573	0,00057505	0,00066705	0,00077378	0,00089759
218	0,00042735	0,00049573	0,00057505	0,00066705	0,00077378	0,00089759
219	0,00042735	0,00049573	0,00057505	0,00066705	0,00077378	0,00089759
220	0,00042735	0,00049573	0,00057505	0,00066705	0,00077378	0,00089759
221	0,00042735	0,00049573	0,00057505	0,00066705	0,00077378	0,00089759
222	0,00042735	0,00049573	0,00057505	0,00066705	0,00077378	0,00089759
223	0,00042735	0,00049573	0,00057505	0,00066705	0,00077378	0,00089759
224	0,00042735	0,00049573	0,00057505	0,00066705	0,00077378	0,00089759
225	0,00042735	0,00049573	0,00057505	0,00066705	0,00077378	0,00089759
226	0,00042735	0,00049573	0,00057505	0,00066705	0,00077378	0,00089759
227	0,00042735	0,00049573	0,00057505	0,00066705	0,00077378	0,00089759
228	0,00042735	0,00049573	0,00057505	0,00066705	0,00077378	0,00089759
229	0,00042735	0,00049573	0,00057505	0,00066705	0,00077378	0,00089759
230	0,00042735	0,00049573	0,00057505	0,00066705	0,00077378	0,00089759
231	0,00042735	0,00049573	0,00057505	0,00066705	0,00077378	0,00089759
232	0,00042735	0,00049573	0,00057505	0,00066705	0,00077378	0,00089759
233	0,00042735	0,00049573	0,00057505	0,00066705	0,00077378	0,00089759
234	0,00042735	0,00049573	0,00057505	0,00066705	0,00077378	0,00089759
235	0,00042735	0,00049573	0,00057505	0,00066705	0,00077378	0,00089759
236	0,00042735	0,00049573	0,00057505	0,00066705	0,00077378	0,00089759
237	0,00042735	0,00049573	0,00057505	0,00066705	0,00077378	0,00089759
238	0,00042735	0,00049573	0,00057505	0,00066705	0,00077378	0,00089759
239	0,00042735	0,00049573	0,00057505	0,00066705	0,00077378	0,00089759
240	0,0002538	0,0002944	0,00034151	0,00039615	0,00045953	0,00053306
241	0,0002538	0,0002944	0,00034151	0,00039615	0,00045953	0,00053306
242	0,0002538	0,0002944	0,00034151	0,00039615	0,00045953	0,00053306
243	0,0002538	0,0002944	0,00034151	0,00039615	0,00045953	0,00053306
244	0,0002538	0,0002944	0,00034151	0,00039615	0,00045953	0,00053306
245	0,0002538	0,0002944	0,00034151	0,00039615	0,00045953	0,00053306
246	0,0002538	0,0002944	0,00034151	0,00039615	0,00045953	0,00053306
247	0,0002538	0,0002944	0,00034151	0,00039615	0,00045953	0,00053306
248	0,0002538	0,0002944	0,00034151	0,00039615	0,00045953	0,00053306
249	0,0002538	0,0002944	0,00034151	0,00039615	0,00045953	0,00053306
250	0,0002538	0,0002944	0,00034151	0,00039615	0,00045953	0,00053306
251	0,0002538	0,0002944	0,00034151	0,00039615	0,00045953	0,00053306
252	0,0002538	0,0002944	0,00034151	0,00039615	0,00045953	0,00053306
253	0,0002538	0,0002944	0,00034151	0,00039615	0,00045953	0,00053306
254	0,0002538	0,0002944	0,00034151	0,00039615	0,00045953	0,00053306
255	0,0002538	0,0002944	0,00034151	0,00039615	0,00045953	0,00053306
256	0,0002538	0,0002944	0,00034151	0,00039615	0,00045953	0,00053306
257	0,0002538	0,0002944	0,00034151	0,00039615	0,00045953	0,00053306
258	0,0002538	0,0002944	0,00034151	0,00039615	0,00045953	0,00053306
259	0,0002538	0,0002944	0,00034151	0,00039615	0,00045953	0,00053306
260	0,0002538	0,0002944	0,00034151	0,00039615	0,00045953	0,00053306
261	0,0002538	0,0002944	0,00034151	0,00039615	0,00045953	0,00053306
262	0,0002538	0,0002944	0,00034151	0,00039615	0,00045953	0,00053306
263	0,0002538	0,0002944	0,00034151	0,00039615	0,00045953	0,00053306
264	0,0002538	0,0002944	0,00034151	0,00039615	0,00045953	0,00053306
265	0,0002538	0,0002944	0,00034151	0,00039615	0,00045953	0,00053306
266	0,0002538	0,0002944	0,00034151	0,00039615	0,00045953	0,00053306
267	0,0002538	0,0002944	0,00034151	0,00039615	0,00045953	0,00053306
268	0,0002538	0,0002944	0,00034151	0,00039615	0,00045953	0,00053306
269	0,0002538	0,0002944	0,00034151	0,00039615	0,00045953	0,00053306
270	0,0002538	0,0002944	0,00034151	0,00039615	0,00045953	0,00053306
271	0,0002538	0,0002944	0,00034151	0,00039615	0,00045953	0,00053306
272	0,0002538	0,0002944	0,00034151	0,00039615	0,00045953	0,00053306
273	0,0002538	0,0002944	0,00034151	0,00039615	0,00045953	0,00053306
274	0,0002538	0,0002944	0,00034151	0,00039615	0,00045953	0,00053306
275	0,0002538	0,0002944	0,00034151	0,00039615	0,00045953	0,00053306
276	0,0002538	0,0002944	0,00034151	0,00039615	0,00045953	0,00053306
277	0,0002538	0,0002944	0,00034151	0,00039615	0,00045953	0,00053306
278	0,0002538	0,0002944	0,00034151	0,00039615	0,00045953	0,00053306
279	0,0002538	0,0002944	0,00034151	0,00039615	0,00045953	0,00053306
280	0,0002538	0,0002944	0,00034151	0,00039615	0,00045953	0,00053306
281	0,0002538	0,0002944	0,00034151	0,00039615	0,00045953	0,00053306
282	0,0002538	0,0002944	0,00034151	0,00039615	0,00045953	0,00053306

283	0,0002538	0,0002944	0,00034151	0,00039615	0,00045953	0,00053306
284	0,0002538	0,0002944	0,00034151	0,00039615	0,00045953	0,00053306
285	0,0002538	0,0002944	0,00034151	0,00039615	0,00045953	0,00053306
286	0,0002538	0,0002944	0,00034151	0,00039615	0,00045953	0,00053306
287	0,0002538	0,0002944	0,00034151	0,00039615	0,00045953	0,00053306
288	0,0002538	0,0002944	0,00034151	0,00039615	0,00045953	0,00053306
289	0,0002538	0,0002944	0,00034151	0,00039615	0,00045953	0,00053306
290	0,0002538	0,0002944	0,00034151	0,00039615	0,00045953	0,00053306
291	0,0002538	0,0002944	0,00034151	0,00039615	0,00045953	0,00053306
292	0,0002538	0,0002944	0,00034151	0,00039615	0,00045953	0,00053306
293	0,0002538	0,0002944	0,00034151	0,00039615	0,00045953	0,00053306
294	0,0002538	0,0002944	0,00034151	0,00039615	0,00045953	0,00053306
295	0,0002538	0,0002944	0,00034151	0,00039615	0,00045953	0,00053306
296	0,0002538	0,0002944	0,00034151	0,00039615	0,00045953	0,00053306
297	0,0002538	0,0002944	0,00034151	0,00039615	0,00045953	0,00053306
298	0,0002538	0,0002944	0,00034151	0,00039615	0,00045953	0,00053306
299	0,0002538	0,0002944	0,00034151	0,00039615	0,00045953	0,00053306
300	0,0002538	0,0002944	0,00034151	0,00039615	0,00045953	0,00053306
301	0,0002538	0,0002944	0,00034151	0,00039615	0,00045953	0,00053306
302	0,0002538	0,0002944	0,00034151	0,00039615	0,00045953	0,00053306
303	0,0002538	0,0002944	0,00034151	0,00039615	0,00045953	0,00053306
304	0,0002538	0,0002944	0,00034151	0,00039615	0,00045953	0,00053306
305	0,0002538	0,0002944	0,00034151	0,00039615	0,00045953	0,00053306
306	0,0002538	0,0002944	0,00034151	0,00039615	0,00045953	0,00053306
307	0,0002538	0,0002944	0,00034151	0,00039615	0,00045953	0,00053306
308	0,0002538	0,0002944	0,00034151	0,00039615	0,00045953	0,00053306
309	0,0002538	0,0002944	0,00034151	0,00039615	0,00045953	0,00053306
310	0,0002538	0,0002944	0,00034151	0,00039615	0,00045953	0,00053306
311	0,0002538	0,0002944	0,00034151	0,00039615	0,00045953	0,00053306
312	0,0002538	0,0002944	0,00034151	0,00039615	0,00045953	0,00053306
313	0,0002538	0,0002944	0,00034151	0,00039615	0,00045953	0,00053306
314	0,0002538	0,0002944	0,00034151	0,00039615	0,00045953	0,00053306
315	0,0002538	0,0002944	0,00034151	0,00039615	0,00045953	0,00053306
316	0,0002538	0,0002944	0,00034151	0,00039615	0,00045953	0,00053306
317	0,0002538	0,0002944	0,00034151	0,00039615	0,00045953	0,00053306
318	0,0002538	0,0002944	0,00034151	0,00039615	0,00045953	0,00053306
319	0,0002538	0,0002944	0,00034151	0,00039615	0,00045953	0,00053306
320	0,0002538	0,0002944	0,00034151	0,00039615	0,00045953	0,00053306
321	0,0002538	0,0002944	0,00034151	0,00039615	0,00045953	0,00053306
322	0,0002538	0,0002944	0,00034151	0,00039615	0,00045953	0,00053306
323	0,0002538	0,0002944	0,00034151	0,00039615	0,00045953	0,00053306
324	0,0002538	0,0002944	0,00034151	0,00039615	0,00045953	0,00053306
325	0,0002538	0,0002944	0,00034151	0,00039615	0,00045953	0,00053306
326	0,0002538	0,0002944	0,00034151	0,00039615	0,00045953	0,00053306
327	0,0002538	0,0002944	0,00034151	0,00039615	0,00045953	0,00053306
328	0,0002538	0,0002944	0,00034151	0,00039615	0,00045953	0,00053306
329	0,0002538	0,0002944	0,00034151	0,00039615	0,00045953	0,00053306
330	0,0002538	0,0002944	0,00034151	0,00039615	0,00045953	0,00053306
331	0,0002538	0,0002944	0,00034151	0,00039615	0,00045953	0,00053306
332	0,0002538	0,0002944	0,00034151	0,00039615	0,00045953	0,00053306
333	0,0002538	0,0002944	0,00034151	0,00039615	0,00045953	0,00053306
334	0,0002538	0,0002944	0,00034151	0,00039615	0,00045953	0,00053306
335	0,0002538	0,0002944	0,00034151	0,00039615	0,00045953	0,00053306
336	0,0002538	0,0002944	0,00034151	0,00039615	0,00045953	0,00053306
337	0,0002538	0,0002944	0,00034151	0,00039615	0,00045953	0,00053306
338	0,0002538	0,0002944	0,00034151	0,00039615	0,00045953	0,00053306
339	0,0002538	0,0002944	0,00034151	0,00039615	0,00045953	0,00053306
340	0,0002538	0,0002944	0,00034151	0,00039615	0,00045953	0,00053306
341	0,0002538	0,0002944	0,00034151	0,00039615	0,00045953	0,00053306
342	0,0002538	0,0002944	0,00034151	0,00039615	0,00045953	0,00053306
343	0,0002538	0,0002944	0,00034151	0,00039615	0,00045953	0,00053306
344	0,0002538	0,0002944	0,00034151	0,00039615	0,00045953	0,00053306
345	0,0002538	0,0002944	0,00034151	0,00039615	0,00045953	0,00053306
346	0,0002538	0,0002944	0,00034151	0,00039615	0,00045953	0,00053306
347	0,0002538	0,0002944	0,00034151	0,00039615	0,00045953	0,00053306
348	0,0002538	0,0002944	0,00034151	0,00039615	0,00045953	0,00053306
349	0,0002538	0,0002944	0,00034151	0,00039615	0,00045953	0,00053306
350	0,0002538	0,0002944	0,00034151	0,00039615	0,00045953	0,00053306
351	0,0002538	0,0002944	0,00034151	0,00039615	0,00045953	0,00053306
352	0,0002538	0,0002944	0,00034151	0,00039615	0,00045953	0,00053306
353	0,0002538	0,0002944	0,00034151	0,00039615	0,00045953	0,00053306
354	0,0002538	0,0002944	0,00034151	0,00039615	0,00045953	0,00053306
355	0,0002538	0,0002944	0,00034151	0,00039615	0,00045953	0,00053306
356	0,0002538	0,0002944	0,00034151	0,00039615	0,00045953	0,00053306
357	0,0002538	0,0002944	0,00034151	0,00039615	0,00045953	0,00053306
358	0,0002538	0,0002944	0,00034151	0,00039615	0,00045953	0,00053306
359	0,0002538	0,0002944	0,00034151	0,00039615	0,00045953	0,00053306
360	0,0002538	0,0002944	0,00034151	0,00039615	0,00045953	0,00053306
361	0,00016834	0,00019528	0,00022652	0,00026276	0,00030481	0,00035358
…	…	…	…	…	…	…
1500	0,00016834	0,00019528	0,00022652	0,00026276	0,00030481	0,00035358

Note: Lines 362 to 1499 contain the same values as 361 and 1500 and have been shortened for the online appendix. To run the script, the corresponding lines must be added. Further, the table must be saved in csv format in the working directory and be named “relapse.csv”.
